# Interoceptive Awareness Skills for Emotion Regulation: Theory and Approach of Mindful Awareness in Body-Oriented Therapy (MABT)

**DOI:** 10.3389/fpsyg.2018.00798

**Published:** 2018-05-28

**Authors:** Cynthia J. Price, Carole Hooven

**Affiliations:** School of Nursing, University of Washington, Seattle, WA, United States

**Keywords:** interoception, awareness, emotion, regulation, therapy

## Abstract

Emotion regulation involves a coherent relationship with the self, specifically effective communication between body, mind, and feelings. Effective emotion regulation involves the ability to accurately detect and evaluate cues related to physiological reactions to stressful events, accompanied by appropriate regulation strategies that temper and influence the emotional response. There is compelling evidence demonstrating links between poor or disrupted awareness of sensory information, or interoceptive awareness, and difficulties with emotion regulation. This paper presents a framework, based on psychological and neurobiological research, for understanding how interoceptive awareness facilitates regulation and an integrated sense of self, and thus contributes to health and well-being. A mind-body therapeutic approach called mindful awareness in body-oriented therapy (MABT), uniquely designed to teach fundamental skills of interoceptive awareness, is described. MABT develops the distinct interoceptive awareness capacities of identifying, accessing, and appraising internal bodily signals that are identified in physiological models as the critical components of interoception for emotion regulation. The explanatory model is that the development of these key interoceptive capacities improves sensory (physical and emotional) awareness, reduces distress, and improves regulation. Strategies for teaching and learning interoceptive awareness are not well-developed in mindfulness or psychotherapeutic approaches, particularly important for people who may have difficulty attending to interoceptive awareness due to stress, chronic pain or trauma. To address this issue, MABT provides an individualized protocol for scaffolding interoceptive awareness through a combination of psychoeducation and somatic approaches explicitly addressing difficulties with interoceptive processing. Clinical vignettes are included to provide exemplars of this approach and to highlight key components of the therapeutic process. Results from research are also included to highlight the acceptability, safety, health outcomes, and possible mechanisms underlying the MABT approach.

## Introduction

Emotion theory and understanding have undergone notable shifts as the field of emotion science has developed. Such shifts in theoretical perspectives often appear to travel along a particular continuum that leans toward either body-oriented or mind-oriented explanations. At one end of the continuum it is bodily cues and sensations that are the key source and explanation for emotional experience, at the other it is cognitive processes. Are emotional feelings pre-conscious, arriving fully formed and physically coherent, and then later interpreted by the mind to be named and understood? Or is it the case that cognitive interpretations of the self and context trigger emotional responses that will organize and energize an emotional response, with consciousness of bodily cues and feelings following?

Early science of emotion pointed to a bodily source: a patterned emotional response in the service of survival. The evolutionary model was subsequently modified in embodied theories of emotional experience (James, 1890; Schachter and Singer, 1962) to include the important roles of awareness and interpretation of bodily cues. With a shift, the 20^th^ century saw an emphasis on the role of mind in determining emotion responses, and furthermore in articulating treatment such as cognitive therapy for emotional pain and dysfunction. As theories have shifted between being more bodily-oriented vs. more cognitively-oriented, there has been increased integration and elaboration of the separate perspectives (Izard et al., 1984). Scientists who followed found that locating the origins of organized emotional response in the body provided a foundation for more nuanced and complex models of emotion response and regulation augmented by cognitive activities such as appraisal and sensitivity to internal signals (Damasio, 1999, 2005). Embodiment theorists in philosophy and anthropology wrote about ‘bringing the body back’ into conceptualization of the self (Evans et al., 1991; Csordas, 1994), a view supported recently in neuroscience by interoception models (Craig, 2015) that indicate how the body and mind interact in complex ways to influence each other as they are expressed and understood as emotion.

Interoception is the perception of sensations from inside the body and includes the perception of physical sensations related to internal organ function such as heart beat, respiration, satiety, as well as the autonomic nervous system activity related to emotions (Vaitl, 1996; Cameron, 2001; Craig, 2002; Barrett et al., 2004). Much of these perceptions remain unconscious; what becomes conscious, i.e., interoceptive awareness, involves the processing of inner sensations so that they become available to conscious awareness (Cameron, 2001). There are multiple processes involved in interoception, reflected in conceptual variations of interoceptive awareness across disciplines (Khalsa and Lapidus, 2016; Khalsa et al., 2018), the evolving state of the science, and the emergence of transdisciplinary models to address conceptual and measurement questions (Farb et al., 2015; Khalsa et al., 2018). Importantly to this paper, there is empirical evidence of the connection between interoceptive awareness and regulation of emotion (Craig, 2015). Such research links a lack of interoceptive awareness with emotion disorders (Paulus and Stein, 2010; Khalsa and Lapidus, 2016); and has opened new avenues for working with difficult to treat or intractable emotional disorders, such as depression, post-traumatic stress disorder (PTSD) and substance use disorder (SUD) (Farb et al., 2015). In addition, research on the neurobiological effects of stress has identified neural and physiological changes subsequent to adversity and trauma that influence emotional experience and internal emotion-related processes and awareness (Evans and English, 2002; Lupien et al., 2006; Ellis et al., 2011, 2013; Taylor et al., 2011). For example, physiologic adaptations to persistent or traumatic stress include both autonomic hyper and hypo arousal (for a review, see Taylor et al., 2011). Thus, converging trends in therapeutic practice and neuroscience invite reconsideration of the body, pointing to its central role in emotional experience and regulation.

The purpose of this paper is to present a framework of emotion regulation that highlights the integrative role of interoceptive awareness and ability. Interoceptive awareness is key to identifying internal physiological processes related to affective feeling, and by so-doing is a means of integrating bodily sensations, cognitive processes, and emotional feeling (Craig, 2015). Hence, interoceptive awareness is a window to emotional experience, as well as potentially providing access to important mechanisms of emotion regulation (Khalsa and Lapidus, 2016). This paper has two distinct sections. The first section presents a framework, based on psychological and neurobiological research, for understanding how interoceptive awareness facilitates regulation and an integrated sense of self, and thus contributes to health and well-being. The second section presents a mind-body therapeutic approach called mindful awareness in body-oriented therapy (MABT), uniquely designed to teach interoceptive awareness skills to increase capacity for emotional regulation, expression, and understanding.

## Section I: Conceptual Framework Relating Interoceptive Awareness and Emotion Regulation

Models specific to interoception and stress response (Schulz and Vogele, 2015), neurobiology (Paulus, 2007), and physiology (Craig, 2002) converge to pinpoint interoception as central to emotion experience and regulation. The stress response system (SRS) directs and organizes a complex sequence of physiological activities to respond to stress and thus ensure homeostatic balance for the organism. The detection, interpretation and behavioral integration of these internal activities involve interoception. In particular, this information from the body has, as well, a necessary and central role in emotion experience and regulation (Garfinkel and Critchley, 2013). We describe a framework for understanding how interoceptive ability contributes to emotional awareness and regulation.

### Interoception

Interoception involves the bi-directional communication between bodily sensation and multiple levels of cortical oversight, a process by which information about invisible internal physiological states are communicated to cognitive centers in the brain in order to support physical and emotional well-being, including effective response to stress via emotional awareness and regulation (Craig, 2003; Critchley and Garfinkel, 2017). Interoception can be seen as a precursor and even a blueprint for emotion response (Damasio, 1999). Sensations from the body underlie most if not all of our emotional feelings, particularly those that are most intense, and most basic to survival (Craig, 2002). It has a role in survival, supporting regulated response to sensations related to bodily integrity (e.g., sensations of hunger, temperature, and pain) as well as emotion sensations directed at social integration (e.g., positive emotion, affection, and intimacy) and physical survival (e.g., fear and anger/aggression). Interoceptive awareness – the ability to identify, access, understand, and respond appropriately to the patterns of internal signals – provides a distinct advantage to engage in life challenges and on-going adjustments (Craig, 2015).

### Emotion Regulation

Regulated emotion is attuned and adapted to relevant psychosocial and physical circumstances, optimizing opportunities to function in a restorative and growth-oriented manner (Porges, 2011). This involves marshaling an adaptive, appropriate emotional response that organizes behavior and benefits an individual, attuned to internal personal cues as well as external circumstances (Blair and Raver, 2012). On the other hand, emotion dysregulation involves an emotional response that is out of proportion, erroneous or inappropriate with respect to the stimulus, and ineffective for achieving overall and consistent well-being. There may be benefits to a dysregulated response (e.g., intense aggression may remove the irritant), however, inappropriate or intensity of emotional sensations and responses distinguish dysregulation as problematic to overall health. In its most intense and persistent manifestations, dysregulated emotion can be characteristic of diagnosable anxiety, depression, and aggressive disorders as well as PTSD.

At a deeper level, emotion regulation involves a coherent relationship with the self, specifically effective communication between body, thoughts, and feelings. It implies tolerance and understanding of signals from the body and the related cognitive attributions. It also implies having the capacity to positively manage challenging sensations and related behavioral responses, such as behaviors or decisions to moderate, suppress or change signals toward a desired end. From an embodiment perspective, the accurate detection and evaluation of cues related to physiological reactions is accompanied by appropriate regulation strategies that temper and influence the emotional response. Optimally, emotional regulation confers benefits in terms of health, well-being, social connection, and competence with life tasks.

### Stress Response System (SRS), Interoception and Emotional Regulation

Being responsive to interoceptive information allows an individual to be aware of an emotion cue early, and therefore to process, interpret and strategize at the onset of stressful events. There is a complex relationship between interoception and stress (Schulz and Vogele, 2015) as both processes reflect the neurological communication between the central nervous system (CNS) and peripheral nervous system (PNS), which is critical to mobilizing the organism for homeostasis and survival, and both are shaped by key interactions with the environment. For these systems, the bi-directional communication between the CNS and PNS involves interoception, perception, and processing of internal bodily states that are transmitted to brain, and involves activated stress processes that are transmitted from the CNS to the peripheral system as well as to metabolic and immunologic functions via neural and endocrine pathways.

General stress models, such as the Allostatic Load model, posit that a stressful environment leads to a dysregulation of the SRS (Del Giudice et al., 2011; Ellis et al., 2011). The SRS codes and filters information from the environment to prepare the body to respond to threats to its equilibrium. The SRS involves several subsystems (SNS; PNS; HPA) each with patterns of response to stress, constituting a primary integrative pathway through which psychosocial environmental factors are transmuted into behavioral, autonomic and immunologic adaptation, or pathology. Dysregulation of the SRS is typically initially reflected in hyper-responsivity and causes wear and tear on physical, mental, and emotional regulatory systems (Del Giudice et al., 2011; Ellis et al., 2011; Blair and Raver, 2012). However, over time, the SRS system may become down-regulated and hence *less* sensitive and responsive to cues, marked by insensitivity to internal states and their causes. Both hyper and hypo sensitivity affects the relationship with the body and emotions: hyper vigilance is associated with overly reactive responses and negative, possibly inaccurate, interpretations; conversely buffered responsivity is less informed and engaged, and therefore less likely to respond when responding is called for. Hence, the excessive and/or unrelenting demands from a difficult environment can get ‘under the skin’ and change a person’s physiological response to stress (McEwan and Seeman, 2003; Lupien et al., 2006; Taylor et al., 2011; Ellis et al., 2013). Such exposure to constant stress and the changes described can lead to decreased interoceptive ability that may be a reflection of the noted difficulties in detecting, tolerating, and interpreting cues.

Schulz and Vogele (2015) present a model that integrates stress response and interoception, suggesting that undue stress affects interoceptive awareness by altering the intensity of the internal cues as well as their perception and interpretation. Thus, stress may influence multiple levels of interoceptive process. Stress and trauma affect the strength of signals at the most basic levels of interoception, as well as the ability to ‘access’ or tolerate the disturbance, which in turn compromises accurate interpretation of sensations and related decisions regarding behavior. Schulz and Vogele focus their arguments on psychological disorders directly influenced by uncomfortable sensations emanating from the body (e.g., rapid heart rate leading to anxiety; dissociation). In addition, we suggest that their argument for maladaptive emotional response can be applied to dysregulated emotional patterns that have documented associations with maladaptive stress responses such as suicide behaviors, depression and anger management disorder (Hooven et al., 1995; Briere and Jordan, 2009; Anestis et al., 2011).

The Adaptive Calibration Model (Del Giudice et al., 2011; Blair and Raver, 2012; Ellis et al., 2013) allows that the benefits of upregulated or down-regulated stress may be momentarily adaptive. However, difficulty arises when the response that is adaptive to difficult environments remains ‘set’ even when the environment is changed. Set points represent a long-term calibration of the SRS during early life events, resulting in consequential patterns of autonomic and HPA responsivity that are sustained long after the events that precipitated them, and possibly long after they are adaptive (Del Giudice et al., 2011; Pluess, 2015). For instance, the individual with a more reactive, open response to stress, developed in a supportive, safe environment, will be at a disadvantage if they continue to be sensitive and reactive in an adverse situation. There is a ‘sweet spot’ in regulation, between being sufficiently buffered so not to be overwhelmed, but still engaged with the environment (Ogden, 2009). This is the therapeutic window where affect is both tolerable and helpful, i.e., affective responses between hyper and hypo arousal.

For a highly responsive individual, the SRS amplifies the signal coming from the environment and maximizes the chance an individual will be modified by that experience; the costs may include being hypersensitive to social criticism or becoming interrupted or overwhelmed by minor challenging events (Blair and Raver, 2012; Pluess, 2015). On the other hand, chronic stress may result in lower tolerance for physiological response, solidifying a strategy at the physiological level to buffer and defend the organism from activation of the SRS, such as physiological ‘set points’ that buffer signals and protect the organism (Del Giudice et al., 2011; Ellis et al., 2013). The tasks and challenges of coping with a difficult environment can shape the capacity to attune oneself to bodily signals, and even affect the shape and size of those signals themselves. From a survival perspective, it may be preferable to be buffered from an onslaught of environmental insults and the resulting cues to respond, thus protecting the organism from mounting undue, ineffective and eventually deleterious stress responses. By and large, an environment with ‘normal’ or common stressors may lead to moderate and somewhat adaptive buffering of external cues, whereas a nurturing, facilitative environment may render one more ‘open’ to the environment, more in tune with bodily sensations, and more likely to adaptively respond to stimuli (Del Giudice et al., 2011). The downside to buffering is that the capacity to maintain awareness, notice feelings and interpret feelings may likewise be compromised, and may remain so long after the need for protection is resolved. Similarly, an individual open to the effects of their environment may have less ability to withstand prolonged or dramatic difficulties and frustrations when they are encountered.

### Implications for Intervention

The work we have presented thus far places physiologic cues at the center of emotion regulation theory and research, and, by logical extension, places the body at the center of intervention approaches designed to address emotion regulation. Such an intervention approach is particularly relevant for individuals who experience undue stress, physical or psychological pain or trauma. Implicit in models of both emotion regulation and stress described above is the importance of attending to the ways daily stressors, large and small, impact body–mind communication, specifically the ability to attend to and interpret internal signals of stress-related emotion.

Therapeutic approaches designed to re-shape the response to environmental cues to make physiologic responses more knowable, accessible and tolerable, and thus available to aid in regulation, will have to work with the client to adjust their ‘set points’ in ways that facilitate optimal emotional responding within a general set of current and relevant environmental expectations. Thus therapeutic work directed toward emotional tolerance may expand the therapeutic window, or the sweet spot, between hypo and hyperarousal. Such activities gently nudge the client toward greater interoceptive awareness and emotional regulation by incrementally moving them toward therapeutic goals in a safe and conscious manner.

## Section Ii: Mindful Awareness in Body-Oriented Therapy

In this section, we present the MABT approach, explicitly designed for teaching and learning interoceptive awareness. MABT was developed by co-author Cynthia Price in the 1980s in response to the need to integrate somatic and emotional awareness work within body-oriented therapy practice. Drawing from Focusing (Gendlin, 1981), an experiential psychotherapeutic approach that involves attention to the “felt sense” to enhance sensory awareness of emotional experience, the MABT approach teaches interoceptive awareness using the combination of manual (touch-based), mindfulness, and psychoeducational approaches.

Mindful awareness in body-oriented therapy develops the distinct interoceptive awareness capacities of identifying, accessing, and appraising internal bodily signals (Cameron, 2001) that are identified in physiological models as the critical components of interoception for regulation (Craig, 2003). An incremental or staged process for teaching these interoceptive awareness skills is used in the MABT approach (see explanatory model, **Table 1**). Integral to the development of interoceptive awareness is the development of mindfulness, specifically the capacity to be in, and maintain attention to present-moment experience with an attitude of openness, curiosity, and self-compassion (Kabat-Zinn, 1990; Bishop et al., 2004). Mindfulness increases tolerance of one’s thoughts and feelings, particularly uncomfortable ones, and facilitates the unlinking of uncomfortable observations from scripted unregulated responses.

**Table 1:** Mindful awareness in body-oriented therapy (MABT) explanatory model.

Interoceptive awareness component	MABT key processes	Related health outcomes
Awareness	Body literacy	Improved sensory awareness
Access	Training Interoceptive awareness exercises	Reduced distress and improved well-being
Appraisal	Mindful body awareness practice	Improved regulation and resilience

While MABT and other mindfulness approaches involve both bottom–up and top–down processes (Taylor et al., 2010), MABT is unique in its strong focus on bottom–up learning processes involving a focus on sensation guided by the use of touch to support learning interoceptive awareness. Linked to emotion regulation, interoceptive awareness is affected by one’s previous experiences of stress – suggesting that interoceptive processes are one way in which stress can alter the capacity to tune into emotion and hence regulate emotion (Schulz and Vogele, 2015). Even if there is some ability to access interoceptive awareness, the capacity to maintain awareness, or move back and forth between cognitive oversight and bodily awareness may be undeveloped. The gentle, coached MABT approach is thus used to facilitate learning, and also helps to build trust and comfort with the material, slowly increasing sensitivity to internal states and awareness of complex internal responses that can shape awareness, self-understanding, decision making processes, and behavior that underlie regulation. MABT research in community settings demonstrates the feasibility, acceptability, and safety of MABT (Price, 2005, 2006; Price et al., 2007, 2012, 2013; Price and Crowell, 2016). These studies involved samples with co-occurring conditions and extensive trauma histories, highlighting the acceptability of MABT teaching processes among highly distressed populations. This section describes each of the MABT stages and includes a clinical example of the therapeutic processes involved.

## Learning Interoceptive Awareness: Mabt Processes and Clinical Examples

### Awareness

To access awareness of inner body sensation one needs to know how to perceive internal sensations. The ability to do so, however, can be unfamiliar or challenging. This is often due to avoidance of sensations (often characterized as being defended from feeling), or due to derealization/depersonalization, a type of dissociative response that is very common among those with high stress or chronic pain (Zaman et al., 2015), as well as among those with a history of trauma (Herman, 1997; Frewen et al., 2008). Often there is little to no knowledge on the client’s part that there are sensations that could be brought into awareness, as the patterns of conscious attention are so strongly set. Thus there can be multiple types of barriers to overcome that all require the development of fundamental skills of awareness. MABT begins by teaching the clients to identify body sensations, this is called *body literacy*, the ability to identify and articulate sensory experience. The naming of sensation is secondary to experiencing sensations, and the complex and nuanced awareness that sensation conveys may be unnamed, particularly when first encountering new sensory experiences. However, the ability to identify and describe sensation is fundamental for interoceptive awareness as it provides a pathway for relating or associating to the body, and thus facilitates perceived linkages between experiences of sensation (i.e., links between physical and emotional awareness, for example increased muscular tension and anger) and linkages between sensation and environmental triggers.

In MABT, body literacy is taught by asking the client what is noticed in response to physical pressure on an area where there is expected sensation, for example an area of physical tension or apparent discomfort. Physical pressure, through client self-touch or by the therapist on an area of the body (e.g., top of shoulder), can be used to guide client awareness to body sensation. Reflective listening techniques and follow-up questions are used to promote finer descriptions of sensory experience. When a client has difficulty finding words to describe sensation, the therapist provides a list of options to see if any match the client’s experience and may also describe what he or she feels tactilely; this models body literacy and can help to teach the client how to engage in the process.

#### Clinical Example

A client receiving his first session MABT session will be asked about where he holds tension in his body. He says he holds tension in his shoulders. During body literacy training, the therapist will put moderate pressure on the top of the client’s shoulders and ask the client to describe how his shoulders feel. The client says that his shoulders feel “fine.” It is not uncommon, particularly individuals who avoid attention to sensation, to reply without answering the question due to the unfamiliarity of identifying and articulating sensory awareness. The therapist repeats the question with more specificity by asking the client how his shoulders feel in the area being pressed. The client replies that his shoulders feel “tight.” The therapist uses reflective listening, repeating the client’s words to promote deeper attention to the sensation by the client, and then asks if he can describe the tightness – for example the quality of the tightness (e.g., ropey, knotty, etc.). The client, responds saying, “hmm... I guess the tightness actually has a sharpness to it – like a burning sensation.” He then adds, “I never realized how much my shoulders hurt. The longer I pay attention, the more aware I am of how the tightness travels up into my neck and also down between my shoulder blades.” He spontaneously takes some deep breaths and then says “I really don’t like feeling this way – which is why I decided to come see you. I’m just holding on to too much stress, I think.” The therapist says, “You think you’re holding on to too much stress…” The client says, “Yea – I work too much and I don’t know how to let go. I can get pretty worked up.” The therapist says, “You just took a couple deep breaths a minute ago and I noticed that your shoulders relaxed a bit. Did you notice that too?” The client: “Not in my shoulders, but I feel a little more relaxed overall.” The therapist: “Good noticing and I’m glad to hear that.”

The therapist continues in the session to ask the client to describe sensation in various places (back, arms, legs, etc.) in order to help the client to attend to sensory awareness and to increase awareness of where he holds tension and what that feels like. The take-home practice focuses on the client practicing this on his own, for example putting pressure on his neck and shoulders and noticing the related sensations in his body. He is encouraged to take deep breaths if the area feels tight and to notice how his body and his shoulders feel when he focuses on breathing deeply. Being more aware of sensation – and the quality of sensation (reflected in how one might describe it) – helps the client to pay attention to bodily experience and may stimulate self-awareness and behavior change (i.e., self-care). In this clinical example, the client came into the 2^nd^ session saying that his take home practice (which he did twice daily, once at work and once after arriving home in the evening) helped to keep the tension from increasing throughout the day and that he was in a better mood in the evenings. He said, “I didn’t realize that my body can tell me how I’m feeling! I guess I need to learn to listen to it more…”

The identification of sensory awareness is used in all aspects of subsequent interoceptive training and practice, as it is the fundamental perception of sensation. The ability to identify sensations is also necessary for engaging in the other aspects of interoceptive awareness (access, sustained attention, and appraisal). Verbally identifying and describing sensory experience facilitates awareness of the links between physical and emotional sensations and the internal cues related to one’s individual responses to stress. Importantly, participant verbalization of sensory experience in the sessions ensures that the therapist is informed about client experience and this helps the therapist to guide the educational and therapeutic process.

Integral to MABT, is a take-home practice. At the end of each session the client/therapist collaboratively come up with the home practice for the interim week based on the session (what was learned), what is most helpful for the client, and what can be feasibly practiced (see **Table 2** MABT Key Components). Client self-touch is used to facilitate the ability to engage in interoceptive awareness at home. Practice is critical for integration of interoceptive awareness skills into daily life. With practice, the client can develop comfort bringing mindful attention to the body and be responsive to interoceptive signals, thus facilitating the recalibration of the SRS maladaptive ‘set-points’ that underlie regulation.

**Table 2:** MABT key interoceptive training processes.

Awareness – stage 1 Body literacy	Access – stage 2 Interoceptive awareness exercises	Appraisal – stage 3 Mindful body awareness practice
Identify body sensations	Breath flow exercise	Capacity to sustain awareness
Articulate body sensations	Tissue softening exercise	Noticing internal shifts
	Internal body attention practice	Re/appraisal based on experiential awareness and insight
Take home practice	Take home practice	Take home practice

### Accessing

The next step in the development of interoceptive awareness is learning to bring attention to inner body experience. This involves learning to focus attention *inside* the body. Since this is often an unfamiliar concept, we teach multiple strategies to provide different experiences and pathways for accessing interoceptive experience. These strategies include: (a) attending to and feeling the sensation and flow of exhaled breath through the body, (b) using intention to feel the softening of areas of muscular tension, and (c) bringing attention to a specific area of internal body (e.g., inside chest, shoulder girdle, abdomen, etc.) We begin with exercises that focus on the movement of breath (strategy a) and intentionally attending to softening in an area that is holding tension (strategy b). These exercises, directed by the therapist, create the initial experience of *feeling* internal sensation, similar to the mindfulness meditation practice of attending to the sensations of breathing. Then, we teach the client to bring mindful attention inside a specific internal space in the body (strategy c). To do this, the therapist provides verbal and tactile guidance to promote the client’s mindful attention to a specific area of the inner body; typically we start with the upper chest as it is a relatively easy area to access and then move to areas that may be more problematic for the client (e.g., an area of discomfort). For all these initial accessing strategies, the therapist assesses whether or not the client is successful in bringing attention to the regions of the body and processes used (e.g., flow of breath), and whether more instruction is needed. This assessment thus guides the therapist’s teaching strategies and attention to potential challenges the client may experience in learning to access interoceptive awareness. These various exercises often become well-used strategies for self-care that are incorporated into daily life to facilitate self-care and regulation, as found in numerous MABT studies highlighting the frequent use of MABT skills in daily life and the perceived helpfulness of these skills/practices (Price, 2005; Price et al., 2011, 2012; Price and Smith-DiJulio, 2016).

#### Clinical Example

The ability to access interoceptive awareness varies greatly from person to person; for some it is relatively easy and little guidance is needed and for others, it can take training and practice. This example is of a client for whom access is challenging and describes the process of disengagement and reengagement that is typical in the learning process with clients for whom the SRS system is downregulated, reflecting a lack of awareness and tolerance for experiencing internal states. The client is a 40-year-old woman with chronic low back pain and depressed mood. She naturally avoids and distracts herself from her pain as much as possible as a coping mechanism to help her function throughout the day. In the past she took pain management classes that were also focused on distraction techniques. She is coming to MABT sessions to learn new ways to relate to pain because her pain levels have remained constant and her ability to manage the pain has decreased, causing her to feel easily irritated, depressed, and to increase use of pain medications. She describes herself as someone who puts others first and has trouble taking time for herself or to attend to her emotional needs; that she is just focused on getting through the day and taking care of her family.

It is the client’s 4^th^ MABT session. In prior sessions she has been introduced to various exercises focused on accessing interoceptive awareness. In this session the aim is to facilitate her ability to bring her awareness into her low back region to increase interaction with, and gain information about, this region of the body that is the source of her pain and likely related to her depressed mood.

To start, the therapist and client talk together for 20 min about how the client is feeling and about her experience with the MABT home practice. On this particular day the client describes her back pain as moderate, and says she is coping well and managing her work and family life. She describes her success in using deep breathing to help her relax and reduce the build-up of tension throughout the day. However, she feels tentative about using breath to target the painful areas of her low back as she is afraid that this will cause spasms and increased discomfort. To assist her with bringing attention to her low back, the therapist asks the client to lie prone on the treatment table and places her hands around (one hand in back and one hand in front) the area of the client’s low back, to provide the physical focus for the client’s mindful attention. The therapist then offers verbal coaching to guide the client’s attention inward to the area of her low back. The client, after multiple tries, is able to bring her attention to the space inside her torso. But each time, as her attention comes toward her lower back region, she finds herself thinking about something else. The therapist asks her to notice where in her body this shift “out” occurs. The client is able to identify disengagement from mindful attention at the point just below her lower thoracic spine – a bit above the primary location of her pain. In response, the therapist moves her hands to up to the lower thoracic region and asks the client to see if she can rest her attention there. The client is then able to maintain her focus in her body. She relaxes, and the therapist notices a deepening of attention or presence in this area of the body. The therapist asks the client what she notices, and the client describes the sensation in this area of her back as “achy.” The therapist suggests that the client simply continue to attend to this area of her body for a little longer. The client is able to be present with her sensory experience in her back for many more minutes and as she does so, she feels her throat tighten and tears come to her eyes. The therapist asks what she is noticing, and she says “I just feel so sad.” At this point her attention shifts out of her body and she opens her eyes.

The therapist encourages the client to stay with the feelings of sadness and the client is able to do so, crying quietly with her eyes closed. The client explains that she is remembering her brother who died 2 years ago, shortly after the birth of her second child, and how sad she is that he is no longer alive. She says that she’s not had a chance to really mourn: “I feel like I just need cry and let him go. I miss him so much.”

When they move to sit in chairs toward the end of the session, the client reports that the achiness in her back has subsided and she feels stronger somehow. She says that she hadn’t been aware of how much sadness she was holding inside. She says, “I feel like I’ve been doing my best to just keep going after he died. But I think I just didn’t want to feel how bad it hurt to have him gone.” She reflects further on when her pain started and continues: “I’ve been trying my best to ignore my back pain and here I am remembering my brother and how much I miss him.” She wonders out loud about whether her avoidant coping style may further distance her from knowing how she feels about aspects of her life. The client and therapist discuss the challenges of accessing and staying connected to inner experience. The client is encouraged that she was able to bring her inner attention to her lower back without feeling panicky. She realizes that she has not had this experience before and that having the firm touch of the therapist helped her to stay calm and refocus her attention whenever she noticed herself thinking about other things. Intrigued by the new sensory information that suggests a relationship between the sad feelings, the memory and loss of her brother, and her back pain, she is eager to practice this process at home as it did not trigger anxiety (like she experienced in practicing targeted breathing). The therapist asks her if she feels comfortable exploring the sadness on her own and she says she does. Collaboratively they develop a take home practice for the week involving a similar process of bring her attention to her lower back, using a small towel under her back (in lieu of touch) to help focus her attention there.

This clinical vignette is an example of how accessing interoceptive awareness can facilitate engagement with sensations, and links between sensations, that were not previously in awareness and that can be important to increase self-understanding and recovery (in this case, the need to acknowledge, attend to and accept her grief). The somatization of this client’s emotional pain, experienced as back pain, reflect the complex physiological and psychological interactions that can occur with a prolonged maladaptive stress response – in this case presenting as depression.

A number of therapeutic elements were critically important for this client to successfully engage in accessing interoceptive awareness. The first was trust in the client/therapist relationship ∼ which was built by the therapist listening carefully to the client’s experience. The therapist knew from earlier communication that the client could easily feel anxious about encountering her pain. The therapist did not push the client to interoceptively access the area of her low back when it was clear that the client would have difficulty sustaining awareness in this area. Second, it was important to stay within the “therapeutic window” (i.e., stretching into new places without becoming overwhelmed). The therapist assessed that the client was unable to stay connected and to access interoceptive experience below the region of her thoracic spine. In response, the therapist moved her hands and thus the ‘targeted area’ for interoceptive awareness shifted to the region of the body closest to the back pain that the client could *successfully access*. Third, facilitating the client’s ability to interoceptively re-engage (after disengaging or coming “out” of connection with the body) involves the therapist’s ability to assess presence in the body. This is a critical skill needed to teach interoceptive or mindful body awareness practices using MABT, as it allows the therapist to consistently gauge whether the client is attending to inner bodily experience. In this vignette, the therapist assessed disengagement (also known as ‘mindwandering’) (Smallwood and Schooler, 2006) and where in the body disengagement occurred. The therapist accomplished this by noticing when the client’s attention was no longer in her body, typically experienced as an energetic shift that is reflected in a tangible change in tissue quality. The therapist can confirm this by asking the client about her experience. As shown in this vignette, the client was aware her shift “out” of the body. The therapist then facilitated the client’s ability to notice where in the body disengagement occurred, and to “catch” this happening in the moment so that the client learns to refocus attention and reengage in interoceptive access and awareness processes. Learning to return attention to the body is critical for successful engagement in accessing and sustaining interoceptive awareness, and typically improves with practice, and the concomitant ability to tolerate uncomfortable sensations ∼ reflecting a reduction in buffering or protection that underlie SRS set-points. In this example, the client accessed her inner body and noticed the kinesthetic sensation of achiness and with increased presence, the sensation of sadness. The interface with this new but intriguing material, combined with an increased sense of well-being, invoked the client’s curiosity and motivated engagement in take-home practice even when, as in this case, accessing interoceptive awareness presented potential challenges requiring time, skill, and patience.

### Sustaining Awareness

The ability to sustain awareness of inner body sensations in critical for receiving, i.e., noticing or being aware, of sensory information. MABT sessions thus build on the body literacy and access skills already learned, by coaching clients in the practice of maintaining awareness and learning to deepen their attentive presence in the body, as exemplified above. MABT research indicates that individuals are able to increase their capacity to sustain awareness as they receive more coaching and practice in mindful body awareness (stage 3 of the intervention process) (Price and Graham, 2016). Importantly, the ability to sustain awareness is associated with increased awareness of physical and emotional states and the links to behavior and environmental and/or interpersonal stressors (Price and Graham, 2016). Results from this same clinical trial also demonstrate that exposure to stage three of MABT is associated with greater improvements in interoceptive awareness, emotion regulation, and reduced affective distress compared to those who are exposed to only MABT stages 1 or 2, demonstrating the importance of sustained mindful attention and appraisal processes in the MABT approach (Price et al., 2017).

Also, it is in the state of sustained mindful attention that individuals most commonly experience new awareness or insight about themselves or a situation (for example, the new awareness of sadness in vignette above). Insight is understood as a change in consciousness that includes a shift in understanding (Kounios and Beeman, 2014), a psychological process thought to inform well-being in meditation practice (Dahl et al., 2015). Such shifts self-understanding often include new awareness of the links between physical and emotional sensations, involving metacognitive awareness processes (Fernandez-Duque et al., 2000) that underlie cognitive appraisal of bodily experiences (e.g., back pain and grief in vignette above), and appear to be critically important for insight, integration of interoceptive experience into self-understanding (i.e., sense-of-self), and the ability to better regulate emotion (Mehling, 2016; Khalsa et al., 2018).

### Reappraisal

Cognitive reappraisal involves reevaluation of a situation or experience such that our response to the situation or experience is altered (Gross, 2001) and when positive, stressful events or experiences can be reconstrued as meaningful or growthful (Lazarus and Folkman, 1984). Developing the capacity for interoceptive awareness is thought to facilitate positive and adaptive reappraisal processes (Garland et al., 2015), a critical aspect of emotion regulation (Webb et al., 2012). In MABT, the therapist coaches the client to attend to the array of possible accessible sensory experiences in order to facilitate appraisal and reappraisal processes. This includes noticing whether shifts in internal experience occur during the session, and noticing the sensory qualities of these shifts. At the end of the session the client is asked to verbally review the session highlights to facilitate cognitive integration of the session material. This review process also facilitates cognitive reappraisal of session experiences in ways that further motivate continued use of interoceptive awareness practices and integration into daily life (Price and Smith-DiJulio, 2016).

#### Clinical Example

The client is a single woman in her late 30s. She has a history of childhood sexual trauma, and has had extensive psychotherapy to aid in her recovery. She works in an extremely stressful job as an executive at a large company. Easily overwhelmed, she finds herself often anxious and extremely stressed about work demands. The client sought MABT because she her elevated stress was triggering recurrent body memories related to her abuse; these memories were interfering with her sleep and her comfort with intimacy with others. Her sense of disconnection from her body was heightened and she wanted to explore a more somatic therapeutic approach for her self-care.

It is the client’s 6^th^ MABT session. She has a high level of emotional awareness, and is quite facile at accessing interoceptive awareness. However, her practice of MABT skills has been limited, in part due to her long work days and in part due to her long-time pattern of avoiding sensory material as a strategy to protect or buffer her from uncomfortable emotions. At the beginning of this session, the therapist guides her through a seated body scan and the client reports noticing a feeling of heaviness in her abdomen, an area that is often uncomfortable when she is anxious or feeling fearful. The therapist and client agree to focus on interoceptive attention to the client’s abdominal region during the session. The therapist and client continue their therapeutic work on the massage table. The therapist has her hands on either side of the client’s abdomen – one on the front and one on the back – and is able to assess through changes in the client’s tissue quality when the client has successfully dropped her attention into, or accessed, her abdominal region. The therapist asks simple guiding questions to facilitate client attention to the sensations within her abdomen. The client initially notices that her abdominal region feels small and closed. The therapist asks if she is aware of any other sensations. The client says that she is aware of the heaviness she mentioned during the body scan. The therapist asks how she would describe the heaviness. At this point the client’s attention immediately shifts out of presence in her body. She fidgets on the table and says “I’m not in there anymore.” The therapist asks what she’s noticing now and the client says she was thinking about some work event. The therapist asks if she’d like to try again and after hearing “yes,” she coaches her again through the process of returning her attention to her abdominal region. The therapist then coaches the client to sink her attention deeply into the heavy sensation in her abdomen; suggesting that she simply be with herself in this small space, to maintain her attention there without needing to do or change anything. The client is able to maintain attention in her abdominal area for a sustained period (about 15 min). The therapist checks in during this time, asking what is noticed. The client replies, indicating that the space is changing, while maintaining mindful presence in her body. The therapist asks if she can describe how it is changing. The client says that it is bigger and feels somewhat lighter. The therapist, using reflective listening, repeats “it is bigger and lighter.” There is a long pause, after which the client continues by adding, “and there is some yellow, like a stream of sunshine coming in from the side.” The therapist asks what else she is noticing. The client, after a long pause responds, saying, “I feel very peaceful.” The client then adds that it’s been a long time since she’s felt so calm inside. The therapist asks her to notice the entire state of her internal body in this experience of calm and peace. The client responds by saying she feels a sense of continuity from her head to her feet; a sense of being whole. She continues noticing her interoceptive experience and says, in a surprised voice, “I have no worries, it is as though my entire being is calm.” After a couple more minutes, the therapist asks her to maintain this state of calm as she slowly returns from this deep internal place of attention, taking her time to open her eyes.

Once seated, they review the client’s experience to facilitate the client’s cognitive integration of the material. The therapist asks the client to notice how her body is feeling while seated, and the client’s most immediate response is that she feels light and relaxed, that her abdomen feels no heaviness inside – just ‘normal’ and good. She continues to reflect on her experience. She looks up at the therapist she says with tears in her eyes that she is amazed that she was able to stay connected inside for so long – and that this experience gives her a new sense of herself and a new sense of hope. In response to the therapist asking her about what she means by “hope,” she replies: “I really want to feel I can continue to feel my body as a safe place; to not feel so anxious and off-center especially when I’m triggered.” The therapist asks her to again notice and to make a strong mental note of her bodily experience of calm and safety, pointing out that this is an important experience of wholeness and safety, one that is not easily accessible when she is feeling anxious and so all the more important that she know that this is possible for her and that she has the capacity to come back to this peaceful and ‘whole’ experience of her body.

As this example illustrates, to support the client’s *appraisal* of interoceptive awareness, MABT is focused on providing the client with individualized training to gain sufficient comfort and skill accessing interoceptive awareness and sustaining awareness to facilitate noticing experiential shifts during mindful awareness practice. These can be profound fundamental shifts in sense-of-self, as in this case involving both positive physical and emotional shifts that reflect recalibration of the SRS set-points. The client’s experience of somatic well-being and embodiment is a significant shift that facilitates trust of her body (i.e., connecting to her body and her emotions can feel safe). Such a positive experience can motivate an individual to engage in further therapeutic work and can lead to further access to, and development of, inner resources for daily life and increased emotion regulation.

### MABT Description Summary

**FIGURE 1:**
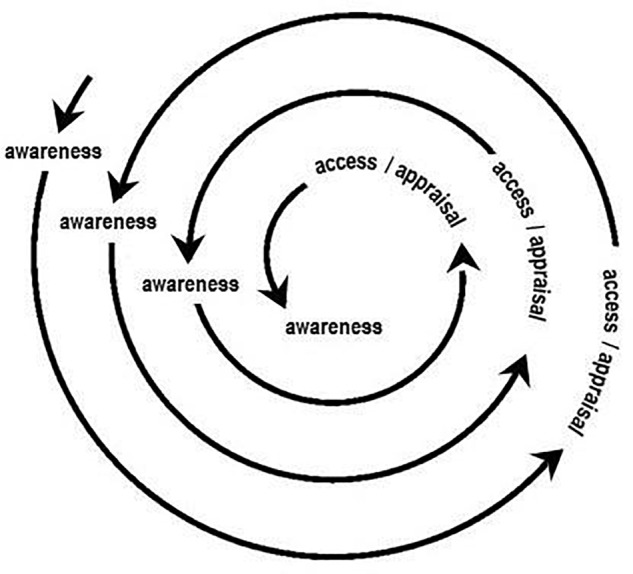
Unfolding interoceptive awareness processes in MABT.

The vignettes illustrate the processes involved in learning interoceptive awareness through MABT. As described, skills are taught incrementally to develop, access, sustain, and appraise interoceptive awareness. As a therapeutic approach, however, MABT is more than simply a strategy for learning interoception. Like other therapeutic approaches, MABT can be provided as the primary modality or in conjunction with other therapeutic or intervention approaches. Thus in clinical care, once basic interoceptive awareness skills are learned, the related therapeutic processes unfold not in a step-by-step linear fashion, but in a way that resembles an ever deepening spiral of awareness, access/sustained attention, and appraisal processes. As illustrated in **Figure 1**, awareness facilitates access, generating deeper awareness, and out of this comes appraisal, which can lead to new awareness and insight. Using MABT skills in daily life to support self-care and bodily connection contributes to the development of life-long practices that promote well-being, embodiment, and emotion regulation.

The MABT learning and therapeutic trajectories vary by individual, thus the teaching and coaching processes must align with the needs of the client. As described, there can be multiple challenges or barriers to bringing attention to the body. These include difficulty knowing where to bring ones attention, the tendency to engage in thinking vs. feeling, a lack of vocabulary to identify or express sensation, not knowing *how* to bring attention into the body, unfamiliarity with maintaining mindful (i.e., present-moment and compassionate) attention in the body, and anxiety related to encountering uncomfortable physical or emotional sensations. Every person has their own ease or challenges learning these skills. Because being with oneself on the inside is inherently an experience of deep self-connection, the client’s sense of safety is paramount. For this reason, an individualized coaching approach allows the therapist to appropriately pace and vary the teaching strategies to support the learning processes and needs of each client. Also, attending to inner bodily sensations can be experienced as a vulnerable activity, particularly if there are challenges involved that touch on feelings of failure due to having trouble engaging in the skills being taught (especially if there is high experiential avoidance), or feelings of fear due to anxiety about contact with unpleasant sensations or emotions (especially if dissociative coping patterns are well-developed). In these types of instances, when engaging in interoceptive awareness can be destabilizing, it is critically important that the therapist has the skills to help the client return to a more stable place, normalizing their experience and serving as a guide to compassionately support the client’s process involving both staying within the ‘therapeutic window’ for any therapeutic work and also recognizing if or when the client may not be ready to pursue this type of therapeutic work. It is thus also important that the therapist has the skills and support to negotiate any related transference and countertransference experiences that may emerge (Pearlman and Saakvitne, 1995; Blackburn and Price, 2007).

In MABT research our experience and findings show that development of interoceptive awareness skills comes more easily to those with familiarity and comfort attending to physical and emotional experience. Nonetheless, research findings from studies with individuals who have with little prior sensory awareness such as populations with chronic illness (HIV) (Price et al., 2013), female veterans with comorbid chronic pain and PTSD (Price et al., 2007), and women in treatment for SUDs who have extensive histories of interpersonal trauma (Price et al., 2012, 2017; Price and Smith-DiJulio, 2016) highlight the accessibility of MABT, and that this relatively brief intervention (delivered once/week across 8 weeks) individuals with little prior sensory awareness can learn interoceptive awareness skills and related practices to increase their ability to emotionally regulate, to manage symptoms of stress, and support their well-being. MABT research demonstrates increased interoceptive awareness skills and concomitant improvements in emotion regulation (self-report and psychophysiology) and reductions in psychological distress for those who receive MABT compared to control and active control conditions (*N* = 187) (Price et al., 2017), suggesting that interoceptive awareness may be the key underlying mechanism supporting these improved health outcomes.

These study findings have important clinical implications, including the potential application of interoceptive awareness training for various health conditions, and the potential for interoceptive awareness skills to be taught and integrated within multiple clinical disciplines (e.g., nursing, social work, psychology, massage therapy, physical therapy, occupational therapy, medicine), settings (e.g., clinics, hospitals, service agencies), and health care conditions (e.g., mental health, chronic pain, chronic illness, and palliative care). The use of touch-based approaches for teaching interoceptive awareness skills, as outlined in this paper, requires appropriate licensure and skills to establish and maintain safety, as well as appropriate training and skills for working with mental health concerns. Relatedly, MABT can be modified so that client self-touch is used in situations which, or by clinicians for whom, touch is not appropriate. It is important to point out that MABT is not specific to those who have difficulties with emotion regulation or for those with serious physical or mental health challenges. Life is inherently stressful (Ellis et al., 2013), and having tools and increased capacity for interoceptive awareness for emotion regulation is useful for most everyone.

Mindful awareness in body-oriented therapy has many features that overlap with mental health approaches that include a focus on mindful attention to the body (such as Hakomi, Sensorimotor Therapy, and Somatic Experiencing). Critically, MABT is primarily focused on teaching therapists *how* to develop client interoceptive awareness skills and thus offers a unique and highly relevant complementary training for therapists in multiple disciplines as well as for psychotherapists interested in incorporating this focus in their practice, whether they have trained in the body-centered approaches like those mentioned above, or in more conventional psychotherapeutic approaches (e.g., cognitive behavior therapy).

## Overall Summary

Individual ability to detect interoceptive signals may be influenced by stress and adverse life experiences that negatively affect willingness, tolerance, interest, and practice with attending to the language of the body. People who have experienced undue stress, chronic pain, or trauma may have ceased to trust or listen to their bodily cues, making it difficult for them to predict their emotional responses and to regulate them. Furthermore, such stress histories appear to affect the magnitude of the interoceptive response, complicating how this important internal information is accessed, processed, and interpreted. The emphasis in MABT on mindful attention to inner body awareness, or interoceptive experience, reconnects the individual to deep bodily states of equilibrium, helping to override and rescript maladaptive stress responses and automatic patterns. The integrated learning processes involved in MABT meld mindfulness practice with active, hands-on coaching, teaching clients to tune-in to the subtleties of physiological sensation and developing interceptive awareness capacity and related appraisal processes. These interoceptive awareness skills facilitate optimal emotional responding and the individual’s ability to process and interpret feelings, or to plan ahead and strategize at the onset of small cues before becoming overwhelmed or entering an unmanageable situation, thus recalibrating the SRS and providing clients with self-care skills critical for emotion regulation.

## Author Contributions

CP and CH made substantial contributions to the conception, writing, final approval and agree that they are both accountable for the contents of this manuscript.

## Conflict of Interest Statement

The authors declare that the research was conducted in the absence of any commercial or financial relationships that could be construed as a potential conflict of interest.
